# Evaluation of the *in vitro* permeation parameters of topical ketoprofen and lidocaine hydrochloride from transdermal Pentravan^®^ products through human skin

**DOI:** 10.3389/fphar.2023.1157977

**Published:** 2023-05-31

**Authors:** Urszula Adamiak-Giera, Anna Nowak, Wiktoria Duchnik, Paula Ossowicz-Rupniewska, Anna Czerkawska, Anna Machoy-Mokrzyńska, Tadeusz Sulikowski, Łukasz Kucharski, Marta Białecka, Adam Klimowicz, Monika Białecka

**Affiliations:** ^1^ Department of Pharmacokinetics and Therapeutic Drug Monitoring, Pomeranian Medical University in Szczecin, Szczecin, Poland; ^2^ Department of Cosmetic and Pharmaceutical Chemistry, Pomeranian Medical University, Szczecin, Poland; ^3^ Department of Chemical Organic Technology and Polymeric Materials, Faculty of Chemical Technology and Engineering, West Pomeranian University of Technology in Szczecin, Szczecin, Poland; ^4^ Department of Experimental and Clinical Pharmacology, Pomeranian Medical University in Szczecin, Szczecin, Poland; ^5^ Department of General and Transplantation Surgery, Pomeranian Medical University, Szczecin, Poland

**Keywords:** penetration skin, vehicles, ketoprofen, lidocaine hydrochloride, Franz diffusion cell, human skin

## Abstract

In the treatment of pain, especially chronic pain, the rule of multimodal therapy applies, based on various painkillers mechanisms of action. The aim of the conducted study was to evaluate the *in vitro* penetration of ketoprofen (KET) and lidocaine hydrochloride (LH) through the human skin from a vehicle with transdermal properties. The results obtained with the use of the Franz chamber showed statistically significantly higher penetration of KET from the transdermal vehicle as compared to commercial preparations. It was also shown that the addition of LH to the transdermal vehicle did not change the amount of KET permeated. The study also compared the penetration of KET and LH by adding various excipients to the transdermal vehicle. Comparing the cumulative mass of KET that penetrated after the 24-h study, it was observed that the significantly highest permeation was found for the vehicle containing additionally Tinctura capsici, then for that containing camphor and ethanol, and the vehicle containing menthol and ethanol as compared to that containing Pentravan^®^ alone. A similar tendency was observed in the case of LH, where the addition of Tinctura capsici, menthol and camphor led to a statistically significant higher penetration. Adding certain drugs such as KET and LH to Pentravan^®^, and substances such as menthol, camphor or capsaicin, can be an interesting alternative to administered enteral drugs especially in the group of patients with multiple diseases and polypragmasy.

## 1 Introduction

Pain is one of the most prevalent health problems, whose control is invariably insufficient despite access to medications with various mechanisms of action (MOA). Safety and effectiveness are two most important aspects of chronic pain pharmacotherapy. Non-steroidal anti-inflammatory drugs (NSAIDs) are the most frequently prescribed group of analgesics, especially among older patients with multimorbidity and polypharmacy. The profile of adverse effects of NSAIDs, including cardiovascular or gastrointestinal complications, significantly limits their application in nociceptive pain treatment (McCarberg and D’Arcy, 2013). On the other hand, guidelines from international and national scientific societies indicate that NSAIDs are first-choice drugs in the treatment of numerous conditions associated with nociceptive pain. Due to clinical difficulties in balancing between the effectiveness and safety of systemic therapy, physicians have started considering changes in the route of administering NSAIDs. An effective therapeutic option in treating pain would be topical preparations which present a very good safety profile and a low risk of adverse effects. It is estimated that the plasma concentration of a NSAID is about 5% following topical administration as compared to oral administration (Derry et al., 2017). Topical and transdermal drug administration have advantages over oral administration. It is characterized by a first-pass metabolism and a delivery system that can be easily terminated at any time. Transdermal absorption of the drug allows the drug to penetrate directly through the stratum corneum, which is the outermost layer of the epidermis. The topically applied drug, after passing through this layer, penetrates into the deeper tissues of the epidermis and dermis. In the next stage, it penetrates into the vascularized skin layer and is then absorbed into the general circulation (Roberts et al., 2021; Mohammed et al., 2022). Out of topical drugs used in chronic pain treatment, the most commonly administered are non-steroid anti-inflammatory drugs, local anesthetics (lidocaine) and capsaicin (natural compound binding to a vanilloid receptor subtype 1—TRPV1, serving as a transmembrane ion channel in neurons). Topical NSAIDs in the form of gel, cream, ointment or patch should be applied to intact skin. These forms are characterized by different transdermal penetration and absorption levels. Furthermore, important aspects associated with topical administration of NSAIDs include: the molecular mass of the applied drug, lipophilicity and the extent of active ingredient permeability. Following topical application, the drugs are absorbed and reach therapeutic concentrations in the surrounding tissues. Serum concentration of a NSAID applied to the skin is considerably lower compared to a drug administered orally, thus the risk of adverse effects is significantly reduced. Topical NSAIDs are currently recommended in treating injury-related muscle pain, and pain and inflammatory symptoms accompanying osteoarthritis of the knee and fine joints in the hand. Compared to oral agents, NSAIDs applied topically to the skin surface present a better safety profile, stemming from lower systemic drug concentrations (Komatsu and Sakurada, 2012). Guidelines from many scientific societies, i.e., the American Academy of Orthopedic Surgeons, the European League Against Rheumatism, the Osteoarthritis Research Society International, the National Institute for Health and Clinical Excellence, recommend using topical agents for pain treatment in osteoarthritis as a first-line option, before using oral drugs (Hochberg et al., 2012; Nelson et al., 2014). Pharmacotherapy optimization with topical NSAIDs involves selecting a suitable active ingredient and vehicle ensuring a beneficial pharmacokinetic profile of the drug, understood as transdermal penetration and absorption into the biophase. The pharmaceutical market offers solely a ready-to-use 2.5% gel with a fixed composition. However, it is possible to produce a compounded medication, as the pharmaceutical raw material base has been extended to include new therapeutic substances for drug compounding (e.g., KET) and new transdermal ointment and cream bases (e.g., Pentravan^®^). Pentravan^®^ is a transdermal liposomal cream base (Chern and Beutler, 1976). Following the dermal application of the vehicle with the active ingredient, special-structure liposomes enclose molecules of the active ingredient and facilitate its transdermal delivery to the circulatory system and subsequently to the biophase (Schwabbauer, 1975; Stamm et al., 1976). Compounding offers the possibility of using a topical medication with an optimum composition and concentration, with a suitable base and absorption enhancers. Personalized treatment of pain with the use of topical drugs may yield optimum effectiveness with a minimal risk of adverse effects, which is particularly important in older patients with multimorbidity and polypharmacy.

The aim of the present study was to assess the *in vitro* human skin permeation of KET and LH from a transdermal base. Furthermore, the concentration and transdermal permeation of KET were compared with the same parameters observed in ready-to-use topical drugs. Based on the testing performed, we determined an optimum composition of the topical preparation, which will offer the most effective permeation of the active substance, and thus the best pharmacological effect.

## 2 Materials and methods

### 2.1 Materials

Pentravan^®^ we, KET, LH, glycerin, menthol, camphor, Tinctura Capsici were purchased from Fagron (St. Paul, MN, United States); 99.5% acetic acid, potassium dihydrogen phosphate, ethanol, methanol, and phosphate-buffered saline (PBS; pH 7.40 ± 0.05) were from Chempur (Piekary Sląskie, Poland), whereas acetonitrile for HPLC was from J.T. Baker (Berlin, Germany). All reagents were of analytical grade.

### 2.2 Skin

Human skin was excised from the abdomen of living patients as a result of plastic surgery. The study was approved by the Ethical Committee of Pomeranian Medical University in Szczecin (KB0012/02/18). The skin of 0.5 mm in thickness was divided into about 2 cm × 2 cm pieces. The fresh human skin was washed in PBS buffer pH 7.4 several times. The skin samples were wrapped in aluminum foil and stored in a freezer at −20°C until use, not longer than 3 months. This frozen storage time was safe to keep skin barrier properties (Badran et al., 2009). On the day of the experiment, the skin samples were slowly thawed at room temperature for 30 min and were hydrated with PBS pH 7.4 (Kuntsche et al., 2008; Simon et al., 2016; Haq et al., 2018).

### 2.3 Vehicles

The study material included six compounded agents for topical use, each with a different composition. The transdermal semi-solid formulations were compounded using Pentravan^®^, a ready-to-use transdermal base. The following medications were added to the vehicle: KET and LH as well as various pharmaceutical raw materials, i.e., glycerol, ethanol, menthol, camphor and tincture of Capsicum (Tinctura capsici). KET was placed in a mortar and mixed with a small amount of the vehicle. Next, the remaining part of the vehicle was added in portions, while gently mixing. LH was ground in a mortar and combined with a portion of the vehicle. Due to a high water content in the vehicle, LH dissolved. Menthol and camphor were micronized with ethanol in a mortar; the vehicle was added in portions. Tinctura capsici was directly introduced into the vehicle. For comparative purposes, two commercial products, labelled as CP1 and CP2, were also analyzed. The composition of individual agents is presented in Table 1.

**TABLE 1:** Composition of the vehicles used in the penetration study.

Ingredient	P-KET	P-KET/LH/GL	P-KET/LH/EtOH	P-KET/LH/EtOH/Met	P-KET/LH/EtOH/Cam	P-KET/LH/Cap
Pentravan^®^	100	100	100	100	100	100
Glycerin	—	5	—	—	—	—
EtOH 96% (v/v)	—		5	5	5	—
Menthol	—		—	10	—	—
Camphor	—		—	—	10	—
Tinctura capsici	—		—	—	—	10
KET	2.5	2.5	2.5	2.5	2.5	2.5
LH	—	4.0	4.0	4.0	4.0	4.0

*The amounts of components are expressed in g.

KET, ketoprofen; LH, lidocaine hydrochloride; P-KET, Pentravan^®^ containing KET; Pentravan^®^ containing KET, LH; P-KET/LH/Gl, Pentravan^®^ containing KET and glycerin; P-KET/LH/EtOH, Pentravan^®^ containing KET, LH and ethanol; P-KET/LH/EtOH/Met, Pentravan^®^ containing KET, LH, ethanol and menthol; P-KET/LH/Cam, Pentravan^®^ containing KET, LH and camphor; P-KET/LH/Cap, Pentravan^®^ containing KET, LH and Tinctura capsici.

### 2.4 Skin permeation studies

The permeation experiments were done by using Franz diffusion cells (Phoenix DB-6, ABL&E-JASCO, Wien, Austria) with diffusion areas of 1 cm^2^. The volume of acceptor chamber was 8 cm^3^ and it was filled with PBS solution (pH 7.4). Each diffusion unit has a constant temperature of 37.0°C ± 0.5°C (Bertges et al., 2020). The acceptor chamber content was stirred with a magnetic stirring bar at the same speed for all cells. The skin was mounted on the donor chamber. The undamaged skin pieces with an even thickness were chosen for experiments. The experiment was carried out for 24 h 1 g of the analyzed vehicle was placed in each donor chamber. The samples of acceptor fluid were collected after 0.5, 1, 2, 3, 4, 5, 8, and 24 h of stirring. After this time, aliquots of the acceptor fluid (0.4 mL) were withdrawn and refilled with fresh buffer of the same pH. KET and LH concentrations in the acceptor phase were determined by HPLC. The cumulative mass (µg·cm^−2^) was calculated based on these concentration. The kinetic profiling of *in vitro* infinite dose steady-state percutaneous absorption has been most often characterized by Fick’s laws of diffusion (Fick, 1885; Higuchi, 1960; Scheuplein and Blank, 1971) as shown in Eqs 1–3. The flux (in µg·cm^−2^·h^−1^) through the human skin into acceptor fluid was determined as the slope of the plot of cumulative mass in the acceptor fluid versus time.

The permeation parameters–fluxes of KET and LH through the skin (J_SS_), the diffusion coefficient (K_P_), and the time required to reach steady-state permeation (lag time–L_T_) were obtained from typically J-shaped profiles by using Eq. 1.

$$A=J_{ss}\left(t-L_{T}\right)$$ (1)

where:A—the cumulative amount of active pharmaceutical ingredient (API) permeating into the receptor compartment [µg IBU·cm^−2^], Jss—the steady-state flux [µg·cm^−2^·h^−1^], t—the time [h], L_T_—the lag time [h].

The steady-state flux was estimated from the slope of the linear portion of the plot of cumulative mass in the acceptor phase over time. The lag time (L_T_) was determined from the x-intercept of the linear part of the plot of cumulative permeation mass in the acceptor phase over time and was used to calculate the diffusion coefficient (K_P_) by using Eq. 2.

$$K_{P}=J_{ss}/C$$ (2)

where:C—the concentration in the donor phase.

The diffusion coefficient (D) was calculated according to Eq. 3:

$$D=l^{2}/6\cdot L_{T}$$ (3)

where:l—the diffusional pathway length.

### 2.5 High-performance liquid chromatography (HPLC)

The concentration of KET and LH in the acceptor fluid was assessed with a liquid chromatography system (Knauer, Berlin, Germany). The HPLC system consists of a model 2600 UV detector, Smartline model 1050 pump, and Smartline model 3950 autosampler with ClarityChrom 2009 software. The detector was operated at 270 nm. The 125 × 4 mm chromatographic column filled with Hypersil ODS (C18), particle size 5 μm, was used. The mobile phase for KET consisted of 0.02 mol/L aqueous potassium dihydrogen phosphate adjusted to pH 2.5 with orthophosphoric acid-acetonitrile-methanol (53/40/7, v/v/v). The spectrophotometric detector was set at 220 nm. For lidocaine hydrochloride acetonitrile, 1% acetic acid, and MeOH (60/30/10, v/v/v) was applied. The spectrophotometric detector was set at 264 nm. The flow rate of 1 mL/min. The column temperature was set at 25°C, and the injection volume was 20 µL.

### 2.6 Statistical analysis

Results are presented as the mean ± standard deviation (SD). A one-way analysis of variance (ANOVA) was used. In the case of the cumulative mass after 24 h permeation and the cumulative mass in the skin, the significance of differences between individual groups was evaluated with Tuckey’s test (*α* < 0.05). A cluster analysis was carried out to determine similarities between all vehicles tested, considering all time points. On this ba-sis, groups of vehicles were presented in the case with a similar penetration. Statistical calculations were done using Statistica 13 PL software (StatSoft, Kraków, Polska).

## 3 Results

**FIGURE 1:**
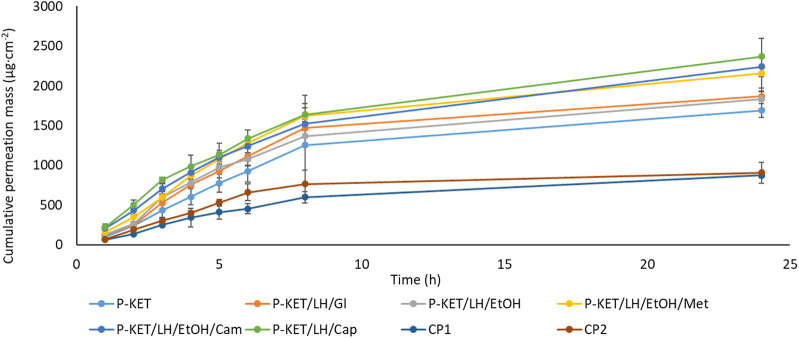
Time course of the cumulative mass of KET during the 24 h permeation. 1 g hydrogels and emulsion/1 cm^3^ of ethanol solution containing a constant concentration of KET was placed in each donor chamber (Mean ± SD, *n* = 3). P-KET, Pentravan^®^ containing KET; P-KET/LH/Gl, Pentravan^®^ containing KET, LH, and glycerin; P-KET/LH/EtOH, Pentravan^®^ containing KET, LH, and ethanol; P-KET/LH/EtOH/Met, Pentravan^®^ containing KET, LH, ethanol, and menthol; P-KET/LH/Cam, Pentravan^®^ containing KET, LH, and camphor; P-KET/LH/Cap, Pentravan^®^ containing KET, LH, and Tinctura capsici; CP1, CP2, commercial products.

**FIGURE 2:**
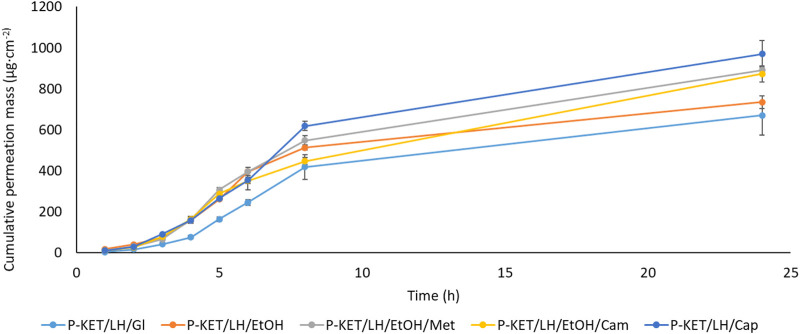
Time course of the cumulative mass of LH during the 24 h permeation. 1 g hydrogels and emulsion/1 cm^3^ of ethanol solution containing a constant concentration of LH was placed in each donor chamber (Mean ± SD, *n* = 3). P-KET/LH/Gl, Pentravan^®^ containing KET, LH, and glycerin; P-KET/LH/EtOH, Pentravan^®^ containing KET, LH, and ethanol; P-KET/LH/EtOH/Met, Pentravan^®^ containing KET, LH, ethanol, and menthol; P-KET/LH/Cam, Pentravan^®^ containing KET, LH, and camphor; P-KET/LH/Cap, Pentravan^®^ containing KET, LH, and Tinctura capsici.

All analyzed drugs permeated the human skin. The cumulative mass of the tested compounds in acceptor fluid, considering all time points, is presented in Figures 1, 2. While the content in the acceptor fluid collected during 24 h permeation is summarized in Table 2. The cumulative mass of the KET from individual vehicles, determined after 24 h of permeation, was as follows: P-KET/LH/Cap > P-KET/LH/EtOH/Cam > P-KET/LH/EtOH/Met > P-KET/LH/GL > P-KET/**LH**/EtOH > P-KET > CP2 and CP1. In the case LH this was P-KET/LH/Cap > P-KET/LH/EtOH/Met > P-KET/LH/EtOH/Cam > P-KET/**LH**/EtOH and P-KET/LH/GL. Both KET and LH penetrated at most from the capsaicin. The cumulative amounts of substances permeated during the 24 h study were 2367.12 ± 233.18 μg·cm^−2^ and 970.66 ± 95.41 μg·cm^−2^, respectively.

**TABLE 2:** Cumulative mass for KET and LH; different letters indicate significant differences *p* < 0.001, *α* = 0.05, mean ± SD, *n* = 3.

Vehicle	Cumulative permeation mass (µg·cm^−2^)	
	KET	LH
P-KET	1691.228 ± 87.313^b^	—
P-KET/LH/GL	1866.066 ± 60.689 ^bc^	670.539 ± 31.813^a^
P-KET/LH/EtOH	1832.381 ± 138.632 ^bc^	734.935 ± 15.774 ^ab^
P-KET/LH/EtOH/Met	2158.041 ± 227.441^cd^	890.891 ± 40.019^c^
P-KET/LH/EtOH/Cam	2238.837 ± 127.450^cd^	872.353 ± 66.018^bc^
P-KET/LH/Cap	2367.118 ± 233.183^d^	970.665 ± 95.413^c^
CP1	871.690 ± 34.320^a^	—
CP2	905.712 ± 128.838^a^	—

KET, ketoprofen; LH, lidocaine hydrochloride; P-KET, Pentravan^®^ containing KET; P-KET/LH/Gl, Pentravan^®^ containing KET, LH**,** and glycerine; P-KET/LH/EtOH, Pentravan^®^ containing KET, LH, and ethanol; P-KET/LH/EtOH/Met, Pentravan^®^ containing KET, LH, ethanol, and menthol; P-KET/LH/Cam, Pentravan^®^ containing KET, LH, and camphor; P-KET/LH/Cap, Pentravan^®^ containing KET, LH, and Tinctura capsica; CP1, CP2, commercial products.

The statistically significant difference was estimated by ANOVA using the Tuckey’s test.

a, b, c, d = different letters indicate significant differences.

**FIGURE 3:**
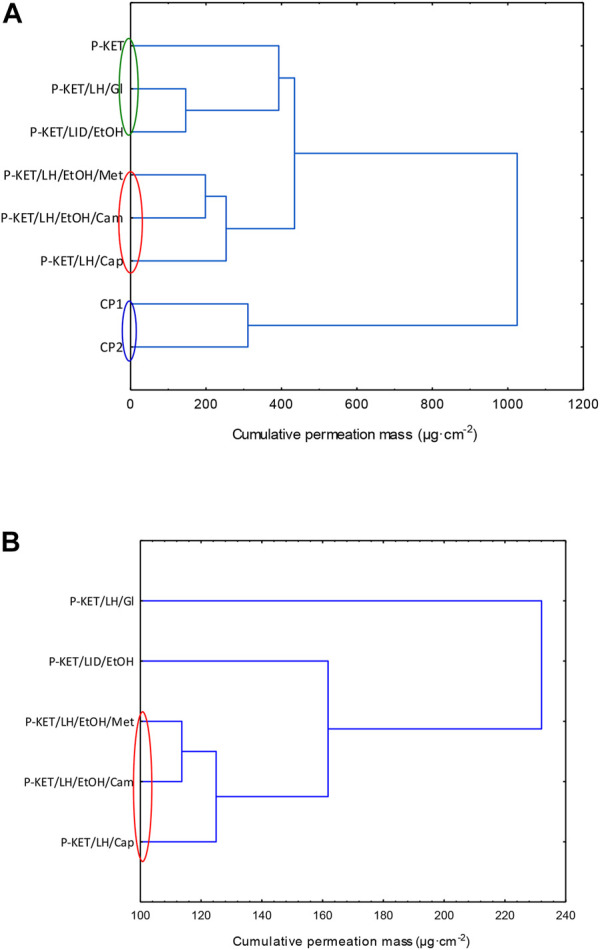
Cluster analysis graph for the mean accumulated mass of KET **(A)** LH **(B)** during the entire 24 h study. The colored circles indicate underwater penetration into individual vehicles. P-KET, Pentravan^®^ containing KET; P-KET/LH/Gl, Pentravan^®^ containing KET, LH, and glycerin; P-KET/LH/EtOH, Pentravan^®^ containing KET, LH, and ethanol; P-KET/LH/EtOH/Met, Pentravan^®^ containing KET, LH, ethanol, and menthol; P-KET/LH/Cam, Pentravan^®^ containing KET, LH, and camphor; P-KET/LH/Cap, Pentravan^®^ containing KET, LH, and Tinctura capsici; CP1, CP2, commercial products.

In general, the permeation of the drugs from the analyzed vehicles containing capsaicin, menthol, and camphor was similar, which was also confirmed by the cluster analysis test (Figure 3). For these three absorption promoters, no statistically significant differences were found in the penetration of both KET and LH (Table 2).

The higher penetration of KET from Pentravan^®^ was observed in the case of preparations containing the absorption promoters, which was also confirmed by the cluster analysis test. This diagram shows the similarity between the various vehicles, that form separate groups marked with colored circles. In the case of KET, the highest and at the same time statistically similar penetration was observed for the vehicle with the addition of menthol, camphor and Tinactura capsici (red circle). The other two groups were characterized by lower penetration, while the group marked with the blue circle had the lowest mean cumulative KET mass after 24 h of penetration. Similar results were seen with LH (red circle)—Figure 3.

The results of the *in vitro* efficiency permeation experiments related to KET and LH are summarized in Table 3. The permeation parameters such as flux, apparent permeability coefficient, lag time, diffusion coefficient in the skin, skin partition coefficient, percent drug permeated after 24 h, and enhancement factor were calculated. The enhancement factor was determined for the active substance—KET. A formulation containing only KET and vehicle—Pentravan^®^ (P-KET) was selected as the reference. There was a clear difference in the steady state flux depending on the vehicle used or the presence of penetration enhancers. In the case of KET, the penetration parameters of the obtained formulations were compared with two commercial preparations (CP1 and CP2). Both commercial formulations showed markedly lower transdermal flux than the Pentravan^®^—based formulations. Moreover, this parameter increases significantly when penetration enhancers are used. The highest value (297.940 ± 70.962 μg cm^−2^ h^−1^) was obtained using Tincture capsici (P-KET/LH/Cap) as an enhancer. In other cases, these values were respectively 276.320 ± 22.324 μg cm^−2^ h^−1^ for P-KET/LH/EtOH/Met, 275.320 ± 42.954 μg cm^−2^ h^−1^ for P-KET/LH/EtOH/Cam, 268.860 ± 15.380 μg cm^−2^ h^−1^ for P-KET/LH/EtOH, 254.380 ± 15.417 μg cm^−2^ h^−1^ for P-KET/LH/GL and 197.110 ± 34.093 μg cm^−2^ h^−1^ for P-KET. The values for the commercial preparations were 1.79 and 1.55 times lower than those for P-KET. The permeability coefficient (K_P_), a quantitative measure of the rate at which a molecule can cross the skin, was also determined. K_P_ is composed of factors related to both the drug and the barrier and their interaction. This parameter eliminates the effect of compound concentration. For the tested formulations, K_P_ values were from 4.402 ∙ 10^3^ cm/h for CP-1 to 11.918 ∙ 10^3^ cm/h for P-KET/LH/Cap. The determined lag time for KET was below 0, which means that the substance is immediately released from the formulation, and its penetration through the skin is immediately noted. Unfortunately, for this reason, it was not possible to determine parameters such as D (diffusion coefficient) and K_m_ (skin partition coefficient) for KET because they depend on lag time. In addition, the percentage of the applied dose was designated. It has been shown that these values range from 3.49% for the commercial product CP-1 and increase by 1.04 times for CP-2, 1.94 for P-KET, 2.10 for P-KET/LH/EtOH, and 2.14 for P-KET/LH/GL, 2.47 for P-KET/LH/EtOH/Met, 2.57 for P-KET/LH/EtOH/Cam to 2.72 for P-KET/LH/Cap. Thus, it confirms that the highest increase in KET permeation was obtained using ethanol and capsaicin (Tincture capsici) as permeation promoters.

**TABLE 3:** Permeation parameters of KET and LH from different Pentravan^®^ formulations through human skin.

Vehicle	J_SS_, μg cm^−2^ h^−1^	K_P_∙10^3^, cm/h	L_T_, h	D∙10^4^, cm^2^/h	K_m_	Q%_24 h_	EF
KET							
P-KET	197.110 ± 34.093	7.884 ± 1.364	a	a	a	6.765 ± 0.349	1.00
P-KET/LH/GL	254.380 ± 15.417	10.175 ± 0.617	a	a	a	7.464 ± 0.243	1.10
P-KET/LH/EtOH	268.860 ± 15.380	10.754 ± 0.615	a	a	a	7.330 ± 0.555	1.08
P-KET/LH/EtOH/Met	276.320 ± 22.324	11.053 ± 0.893	a	a	a	8.632 ± 0.910	1.28
P-KET/LH/EtOH/Cam	275.320 ± 42.954	11.013 ± 1.718	a	a	a	8.955 ± 0.510	1.32
P-KET/LH/Cap	297.940 ± 70.962	11.918 ± 2.838	a	a	a	9.468 ± 0.933	1.40
CP1	110.060 ± 43.602	4.402 ± 1.744	a	a	a	3.487 ± 0.137	0.52
CP2	126.720 ± 14.646	5.069 ± 0.586	a	a	a	3.623 ± 0.435	0.53
LH							
P-KET	—	—	—	—	—	—	—
P-KET/LH/GL	69.907 ± 3.283	1.748 ± 0.082	1.610 ± 0.064	2.589 ± 0.104	0.338 ± 0.028	1.676 ± 0.080	—
P-KET/LH/EtOH	106.32 ± 11.101	2.658 ± 0.278	1.401 ± 0.275	2.973 ± 0.646	0.447 ± 0.132	1.837 ± 0.039	—
P-KET/LH/EtOH/Met	113.840 ± 10.462	2.846 ± 0.262	1.454 ± 0.094	2.866 ± 0.190	0.496 ± 0.076	2.227 ± 0.100	—
P-KET/LH/EtOH/Cam	94.292 ± 5.871	2.357 ± 0.147	1.159 ± 0.103	3.597 ± 0.313	0.327 ± 0.048	2.181 ± 0.165	—
P-KET/LH/Cap	89.313 ± 2.397	2.223 ± 0.060	1.065 ± 0.061	3.911 ± 0.220	0.285 ± 0.022	2.427 ± 0.239	—
CP1	—	—	—	—	—	—	—
CP2	—	—	—	—	—	—	—

J_SS_, steady-state flux; K_P_, permeability coefficient; L_T_, lag time; D, diffusion coefficient; K_m_, skin partition coefficient; Q, the percentage of the applied dose; EF, enhancement factor; * L_T_ < 0. a = not determined.

A completely different permeation profile was observed for LH. In particular, it should be noted that this compound requires some delay for its enhanced permeation to begin. In this case, the effects of different promoters on LH permeation were compared. The lag time was changed by introducing a different permeation enhancer. The lowest L_T_ (1.065 ± 0.061 h) was obtained for P-KET/LH/Cap, while the highest (1.610 ± 0.064 h) for P-KET/LH/GL. The diffusion coefficient in the skin was approximately 2.589 ± 0.104 and 3.911 ± 0.220 cm^2^ h^−1^ for P-KET/LH/GL and P-KET/LH/Cap, respectively (Table 3). Another important parameter is K_m_, which describes the drug’s ability to escape from the solution and move into the outermost layers of the SC. It is defined as the equilibrium solubility of the drug in the SC relative to its solubility in the vehicle. For LH, K_m_ values ranged from 0.285 ± 0.022 to 0.496 ± 0.076 for P-KET/LH/Cap and P-KET/LH/EtOH/Met, respectively. Considering all the release parameters, the highest permeability of LH was obtained, similar to KET, using Tincture capsici as the penetration promoter.

**FIGURE 4:**
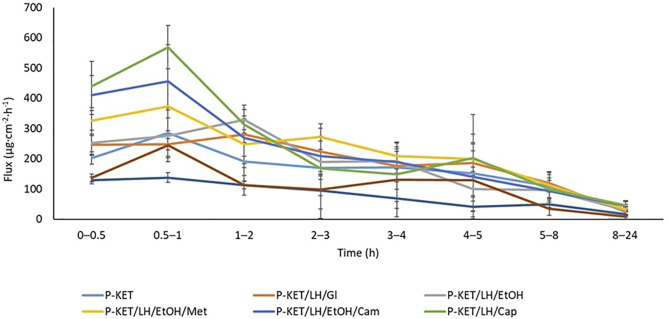
The permeation rate of KET during the 24 h permeation; *α* = 0.05 (Mean ± SD, *n* = 3). P-KET, Pentravan^®^ containing KET; P-KET/LH/Gl, Pentravan^®^ containing KET, LH, and glycerin; P-KET/LH/EtOH, Pentravan^®^ containing KET, LH, and ethanol; P-KET/LH/EtOH/Met, Pentravan^®^ containing KET, LH, ethanol, and menthol; P-KET/LH/Cam, Pentravan^®^ containing KET, LH, and camphor; P-KET/LH/Cap, Pentravan^®^ containing KET, LH, and Tinctura capsici; CP1, CP2, commercial products.

**FIGURE 5:**
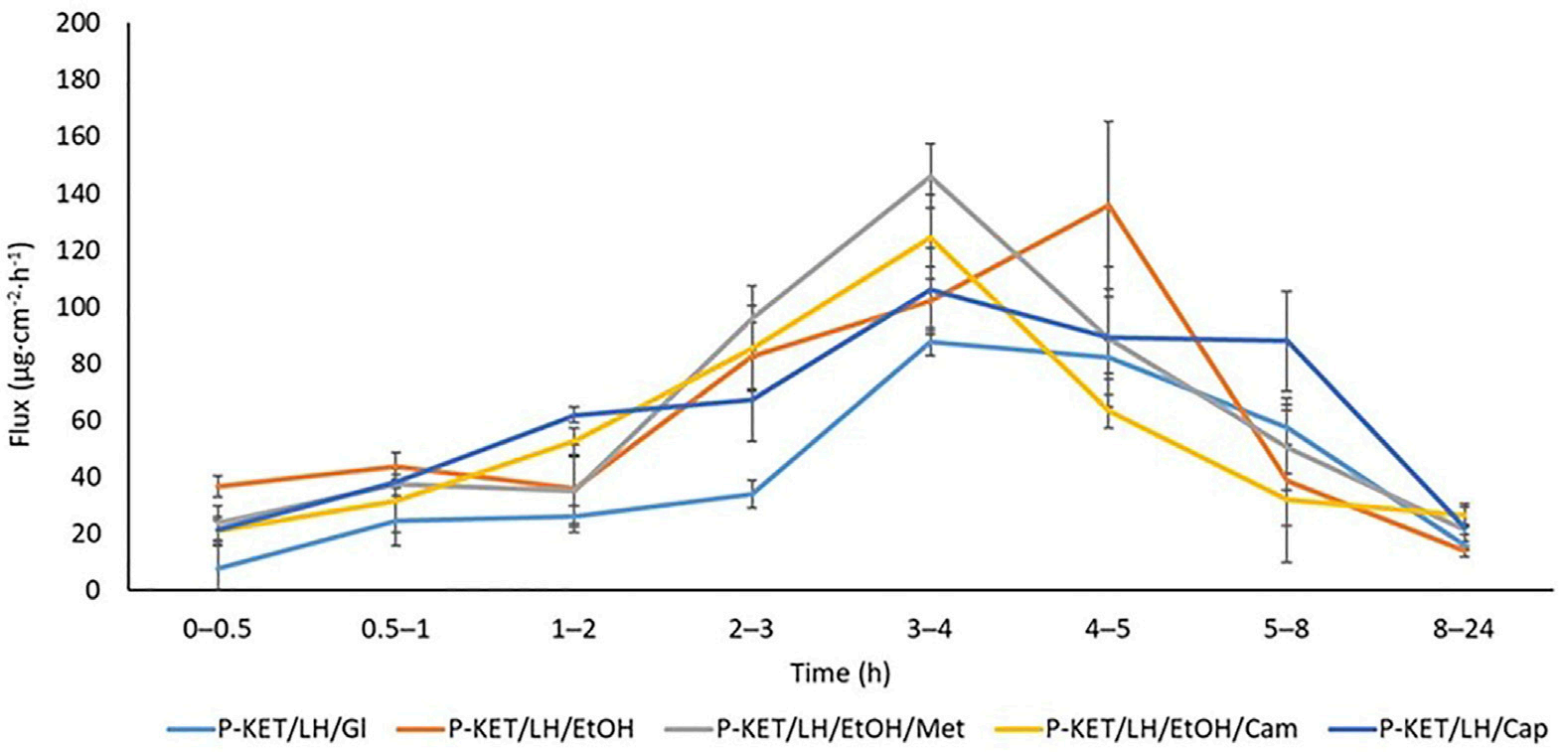
The permeation rate of LH during the 24 h permeation; *α* = 0.05 (Mean ± SD, *n* = 3). P-KET/LH/Gl, Pentravan^®^ containing KET, LH, and glycerin; P-KET/LH/EtOH, Pentravan^®^ containing KET, LH, and ethanol; P-KET/LH/EtOH/Met, Pentravan^®^ containing KET, LH, ethanol, and menthol; P-KET/LH/Cam, Pentravan^®^ containing KET, LH, and camphor; P-KET/LH/Cap, Pentravan^®^ containing KET, LH, and Tinctura capsici.

The permeation rate determined at each time interval is presented in Figures 4, 5. The highest permeation rate to the acceptor fluid was observed in samples collected during the first 2 hours of penetration for KET and between 4–6 h for LH.

## 4 Discussion

The present study assessed the impact of a vehicle and absorption enhancers on *in vitro* permeation of topically applied KET and LH using Franz diffusion cells. The donor phase comprised the Pentravan^®^ transdermal base with KET at a constant concentration of 2.5% and LH at a constant concentration of 4%, with the addition of various pharmaceutical substances (menthol, camphor, Tinctura capsici, ethanol and glycerol). For comparative purposes, we also assessed KET permeation from two commercial products with the same concentration of the active ingredient, without the addition of lidocaine. Our study showed that KET and LH combined with the Pentravan^®^ transdermal base are effectively released from the vehicle and permeate the human skin. It was also demonstrated that the addition of absorption enhancers to the vehicle, such as menthol, camphor or Tinctura capsici, resulted in a significantly higher permeation of the drugs analyzed.

The assessment of *in vitro* penetration is a very important step in selecting a suitable active ingredient and determining the composition of a topical drug vehicle. The drug penetration level might differ depending on the physicochemical properties of the compounds and the vehicle used (Janus et al., 2020; Ossowicz-Rupniewska et al., 2021). Stratum corneum (SC), the outermost layer of the skin, composed mainly of lipids and ceramides, is the main barrier to exogenous therapeutic compounds used transdermally. SC limits the penetration of topical drugs (Kawana et al., 2020) prevents excessive water loss and provides protection against microorganisms, allergens and chemicals (Ossowicz-Rupniewska et al., 2021).

Topical agents containing NSAIDs allow for mitigating pain in the application area, at the same time minimizing the risk of systemic adverse effects. Following the oral administration of NSAIDs, serum concentrations of these drugs reach high values, which significantly increases the risk of severe complications associated with taking these medications, e.g., GI-bleeding, cardio-vascular complications, renal failure and interactions with other simultaneously used drugs. This is particularly important in older patients with multimorbidity and polypharmacy (Komatsu and Sakurada, 2012). To reduce the incidence of adverse effects of NSAIDs, especially when managing pain associated with osteoarthritis of the peripheral joints, drugs from this group (e.g., KET, diclofenac) are used topically. The results of clinical studies point to a high effectiveness of topical KET as compared to other drugs form this group. This may be related to its lower molecular mass compared to other transdermal NSAIDs and high lipophilicity, which results in good transdermal drug penetration into inflamed tissues (Yano et al., 1986).

KET is a non-steroid anti-inflammatory drug with analgesic and antipyretic properties (Kuczyńska and Nieradko-Iwanicka, 2021). The MOA of KET consists in inhibiting the activity of cyclooxygenase COX-1 and COX-2. KET administered cutaneously has been used in the treatment of rheumatic diseases of the motor system, especially osteoarthritis of the peripheral joints and the spine, where its topical application may successfully alleviate post-traumatic lesions or tissue inflammation. On the other hand, most of the ready-to-use drugs available in the pharmaceutical market present poor transdermal penetration, thus producing a weaker analgesic effect. Therefore, apart from selecting appropriate active ingredients with an analgesic effect, it seems valid to search for new drug formulations which would ensure better release and penetration through the selectively permeable SC, and thus result in a higher, long-lasting concentration of the drug in the biophase.

As a rule, chronic pain is managed with a multimodal analgesia approach, which involves simultaneous use of medications with different mechanisms of action (Kocot-Kępska et al., 2021). Unfortunately, many countries lack multi-compound drugs ready for topical application. Selection of an appropriate vehicle can play a key role in increasing drug penetration and obtaining an immediate therapeutic effect. Recent years have seen the emergence of different transdermal vehicles with a fixed composition, whose aim is to increase drug penetration. One of such vehicles is Pentravan^®^, an oil-in-water emulsion base (o/w). The main component of Pentravan^®^ is water (62%). Despite the relatively high content of water, the base has the consistency of thick, yellow cream with a pH of 4.0–5.5. Pentravan^®^ also contains the LIPOIL complex, butylated hydroxytoluene, simethicone, urea, potassium sorbate, polyoxyethylene stearate, cetyl alcohol, stearyl alcohol, stearic acid, glycerol monostearate, benzoic acid, poly (acrylic acid) and hydrochloric acid. The base composition also includes transdermal drug delivery enhancers, substances improving skin hydration, preservatives, stabilizers and enhancers of the rheological properties of ointments and creams (Kuntsche et al., 2008). To date, there have been few publications assessing the release of selected drugs from such a modern vehicle as Pentravan^®^. In our previous study, we investigated the impact of this vehicle on the permeation of ibuprofen and its derivatives, i.e., ion-pairs of ibuprofen with new L-valine alkyl esters [ValOR][IBU]. All the ibuprofenates studied as well as pure ibuprofen displayed considerably higher permeation from Pentarvan^®^ as compared to commercial preparations (Ossowicz-Rupniewska et al., 2021). Pentravan^®^ has also been used as a carrier of active ingredients in a study on hormonal therapy, which analyzed vaginal hormonal drug permeation from Pentravan^®^ (Haddad, 2014).

The main aim of the present study was to assess the extent of KET permeation from Pentravan^®^ as compared to commercial preparations available in the market. The results obtained with the use of the Franz cell chamber demonstrated statistically significantly higher permeation of KET from the transdermal vehicle. In contrast, the ready-to-use commercial preparations displayed a considerably lower transdermal flux of this drug compared to preparations based on Pentravan^®^. The cumulative mass after 24 h of permeation was max. 905.712 ± 128.838 μg·cm^−2^ in the case of commercial preparations and 1691.228 ± 87.313 μg·cm^−2^ to 2367.118 ± 233.183 μg·cm^−2^ in the case of Pentravan^®^. Similarly significantly higher permeation was also demonstrated in our previous study which investigated the penetration of ibuprofen and its derivatives from Pentravan^®^. In that study, we also made a comparison with ready-to-use products (Ossowicz-Rupniewska et al., 2021). Better drug permeation from Pentarvan^®^ is most likely due to its composition. Pentravan^®^ is a liposomal cream base with a transdermal mechanism of active ingredient absorption. In this vehicle, lecithin-based liposomes facilitate active ingredient permeation across the layers of the epidermis. Following the dermal application of the cream, liposomes penetrate the SC through intracellular channels to the deeper layer of the epidermal tissue and then to the highly vascularized dermis. Lipids accumulated in the extracellular structure of the epidermis form a route for active ingredient delivery (Bourdon et al., 2016). The present study demonstrated a very good KET permeation from the transdermal vehicle as compared to the ready-to-use commercial gel preparations, which may translate into effective treatment, resulting in a better pharmacological effect.

Recent years have seen more frequent attempts to combine anti-inflammatory drugs with local anesthetics. Therefore, we decided to assess the penetration of LH combined with KET. Lidocaine is a local anesthetic acting through selective blockade of voltage-gated sodium channels in nociceptive nerve fibers. This leads to the inhibition of the generation and propagation of nerve impulses in pathologically stimulated, damaged nerve fibers responsible for the transmission of pain information. The possibility of combining drugs with different mechanisms of action could allow for improving treatment effectiveness, particularly in the case of mixed pain. The combination of KET and lidocaine in our study is an example of a multimodal management of mixed pain, as in most cases nociceptive pain is accompanied by a neuropathic component and *vice versa*. Our study showed that adding LH to the transdermal base did not affect the extent or rate of KET permeation. In all cases, the recorded lag time (Lt) for KET was below 0, which means that the substance was instantly released from the preparation and immediately permeated through the skin. This outcome points not only to KET’s rapid onset of action, but also to the lack of adverse pharmacokinetic interactions between lidocaine and KET at the absorption level. Our assessment of the LH permeation profile demonstrated a significantly delayed absorption of this drug as compared to KET, whose maximum concentration in the acceptor fluid was achieved much earlier. The analysis of the LH mass in the acceptor fluid collected in particular time intervals showed that the highest permeation occurred after 4–6 h, which is very beneficial due to lidocaine’s required local anesthetic effect on the skin and adjacent tissues. The result observed for KET is also satisfactory. The highest levels of this drug in the acceptor fluid were observed in 1–2 h of the study. With NSAIDs, rapid percutaneous absorption is beneficial, as it produces a rapid therapeutic effect. The increased permeation within a shorter period of time causes a more rapid decrease in inflammation of the underlying tissues (Singh and Savoy, 2020).

In the case of topical preparations, it is useful to add various types of absorption enhancers to the vehicle, as these will improve drug penetration. Thus, in our study we attempted to assess the extent of drug permeation in the presence of absorption enhancers and pharmaceutical raw materials (glycerol, ethanol, menthol, camphor and Tinctura Capsisi). It is commonly known that, when added to topical preparations, certain substances like menthol, camphor or capsaicin have an analgesic effect confirmed in experimental and clinical models. Our study demonstrated that both KET and LH hydrochloride (from the preparations analyzed) achieved good permeability across the human skin. When analyzing the cumulative mass of KET absorbed after 24 h of the study, we observed that compared with Pentravan^®^ alone the drug’s permeation was significantly the highest from the vehicle which additionally contained Tinctura capsici (2367.118 ± 233.183 μg·cm^−2^), followed by the one with camphor and ethanol (2238.837 ± 127.450 μg·cm^−2^) and menthol and ethanol (2158.041 ± 227.441 μg·cm^−2^). A similar association was observed in the case of LH, where the addition of Tinctura capsici, menthol and camphor resulted in a statistically significantly higher penetration of the drug, namely, 970.665 ± 95.413 μg·cm^−2^, 890.891 ± 40.019 μg·cm^−2^ and 872.353 ± 66.018 μg·cm^−2^, respectively. The use absorption enhancers, such as menthol, camphor or capsaicin which is the main ingredient of Tinctura capsici, has previously been described in other studies (Jeevan et al., 1997; Degim et al., 1999; Chen et al., 2013; Xie et al., 2016). Terpenes, such as menthol or camphor, are often used as penetration enhancers. Through their specific MOA, these substances undoubtedly have an impact on pharmacokinetic parameters of drugs. The MOA of terpenes mainly involves changes in the SC barrier structure and interactions with intercellular lipids (Nowak et al., 2021). SC is composed of ceramides, which are tightly packed as bilayers due to the high degree of hydrogen bonding. It is likely that the penetration of terpenes into the lipid layers of SC disrupts the hydrogen bonding network. Terpenes loosen the ceramide structure (Aqil et al., 2007), thus increasing drug penetration (Chen et al., 2015; Nowak et al., 2021). Menthol is commonly used in medicine as a penetration enhancer due to its high effectiveness and relative safety (Wan et al., 2015; Wang and Meng, 2017; Huang et al., 2019). Studies have shown that camphor causes relatively minor skin irritation (Xie et al., 2016). In turn, the MOA of capsaicin, which is classified as an alkaloid (Reyes-Escogido et al., 2011), involves disrupting the lipid layer and decreasing the diffusion resistance between cells (Degim et al., 1999).

Another aspect related to the use of the absorption enhancers studied, i.e., menthol, camphor or capsaicin, is their beneficial (e.g., analgesic) effect, which is particularly important in extending the therapeutic scope of the preparations used and, presumably, reducing the risk of adverse effects associated with topical drug application. For instance, capsaicin is a potent vasodilator and a selective agonist of TRPV1 which is a key receptor in the modulation of nociceptive information transmission (Szallasi, 1994). Menthol is an anesthetic agent with counter-irritant properties. The MOA of this substance involves stimulation of nerve endings transmitting cold and desensitization of nociceptive fibers. As a result, menthol imparts a desensitizing, cooling and antipruritic effect (Galeotti et al., 2002). As for camphor, when applied on skin, it stimulates sensory nerve endings, causes local skin hyperemia and induces a warming sensation, which is beneficial in neuralgia pharmacotherapy. The specific effects of menthol, camphor or capsaicin may be reflected in clinical research which will focus on obtaining a greater analgesic effect and increased availability of the main analgesics, such as ketoprofen, and local anesthetics, such as lidocaine. Our study is in line with the pharmacovigilance system recommended in the EU directives, i.e., the safety monitoring of pharmaceutical products to detect hazards associated with the use of drugs and the elimination or prevention of such hazards. The European Medicines Agency (EMA) has published guidelines on Good Pharmacovigilance Practices (GVP) (www.ema.europa.eu/EMA/54854/2021, n.d.), emphasizing the need to minimize the risk of the treatment applied. These rules also underpin personalized therapy strategies which are tailored to the patient’s individual traits and diseases, thus increasing the chances for effective and safe treatment.

## 5 Conclusion

The use of transdermal medications offers numerous benefits as compared to oral drugs. Cutaneous application may be an effective way of preventing or minimizing the possible adverse effects associated with oral drug administration. Furthermore, the percutaneous penetration of active ingredients can be enhanced by the selection of a suitable vehicle. In our study, we assessed the permeation of KET and LH the Pentravan^®^ transdermal vehicle. We also compared the penetration of KET with commercial products containing the same drug in the same concentration. Our study showed that Pentravan^®^ may be an excellent transdermal vehicle ensuring rapid permeation of analgesic and anti-inflammatory drugs, such as KET. Compared with other preparations available in the market, Pentravan^®^ delivered a higher drug dose. We also attempted to combine KET with LH in this base. We demonstrated that there was no interaction between the drugs at the absorption level. The highest percutaneous permeation of KET was observed already 1 h after application, whereas with LH, the permeation was highest after about 4 h. The use of topical preparations with the composition analyzed may substantially improve the effectiveness and safety of topical pain pharmacotherapy. Furthermore, we observed a significantly higher penetration rate when absorption enhancers, such as menthol, camphor and capsaicin-containing Tinctura capsici, had been added to Pentavan^®^. Another advantage of using these enhancers is their analgesic and antipruritic effect.

It is commonly recognized that the treatment of pain, particularly chronic pain, involves a multimodal approach, employing different MOAs of drugs. It would seem that the same principles could apply to topical formulations used in the management of pain in, e.g., bone and joint diseases. The lack of multidrug formulations with good pharmacokinetic parameters in the pharmaceutical market has motivated the attempts to describe the most beneficial multidrug combinations offering a synergistic effect. The present study showed that the combination of Pentravan^®^ with certain drugs (such as KET and LH) and the addition of substances which act as absorption enhancers, e.g., menthol, camphor or capsaicin, may constitute an interesting alternative for enteral drugs, especially in a group of patients with multimorbidity and polypharmacy.

## Data Availability

The original contributions presented in the study are included in the article/supplementary material, further inquiries can be directed to the corresponding author.
